# Large-scale 3D chromatin reconstruction from chromosomal contacts

**DOI:** 10.1186/s12864-019-5470-2

**Published:** 2019-04-04

**Authors:** Yanlin Zhang, Weiwei Liu, Yu Lin, Yen Kaow Ng, Shuaicheng Li

**Affiliations:** 1 Department of Computer Science, City University of Hong Kong, 83 Tat Chee Avenue, Kowloon, Hong Kong SAR; 2 Research School of Computer Science, the Australian National University, Canberra, Australia; 3 Department of Computer Science, Faculty of Information and Communication Technology, Universiti Tunku Abdul Rahman, Kampar, Malaysia

**Keywords:** Hi-C, 3D chromosome structure, Multidimensional scaling, Chromosome conformation capture, 3D genome

## Abstract

**Background:**

Recent advances in genome analysis have established that chromatin has preferred 3D conformations, which bring distant loci into contact. Identifying these contacts is important for us to understand possible interactions between these loci. This has motivated the creation of the Hi-C technology, which detects long-range chromosomal interactions. Distance geometry-based algorithms, such as ChromSDE and ShRec3D, have been able to utilize Hi-C data to infer 3D chromosomal structures. However, these algorithms, being matrix-based, are space- and time-consuming on very large datasets. A human genome of 100 kilobase resolution would involve ∼30,000 loci, requiring gigabytes just in storing the matrices.

**Results:**

We propose a succinct representation of the distance matrices which tremendously reduces the space requirement. We give a complete solution, called SuperRec, for the inference of chromosomal structures from Hi-C data, through iterative solving the large-scale weighted multidimensional scaling problem.

**Conclusions:**

SuperRec runs faster than earlier systems without compromising on result accuracy. The SuperRec package can be obtained from http://www.cs.cityu.edu.hk/~shuaicli/SuperRec.

**Electronic supplementary material:**

The online version of this article (10.1186/s12864-019-5470-2) contains supplementary material, which is available to authorized users.

## Backgound

Genome-wide sequencing studies, such as the Human Genome Project (HGP) [1, 2], have deciphered the genomic sequences of humans. We are now in a position to reconstruct the 3D structure of the genome, that is, the conformations of the chromosomes within the nucleus. This will further our understanding of chromosomal interactions.

Recent discoveries through imaging analysis revealed that, while chromosome conformations may vary from cell to cell, they are not random [3, 4]. Hotspots of interactions and transcriptions are unevenly distributed; transcriptionally inactive segments prefer locations such as on nuclear periphery, around nucleoli, or at nuclear substructures [5–16]. These observations all point to the fact that gene expressions are highly associated with the chromatin structure.

However, since imaging techniques are not yet able to achieve high enough resolutions for genome-wide studies, researchers have sought to reconstruct the chromatin structure from knowledge of the interactions between genomic loci [17–23]. Valuable insights on gene regulations, genome translocations, copy number variations, genome stability, *etc.*, have been derived, owing to the success of these methods [24–31]. Among the technologies for capturing interaction information, one called Hi-C has been used prominently. The method produces a matrix, called a *contact map*, which stores the normalized frequencies between all pairs of genome loci (also referred to in the literature as *bins*, *regions* or *windows*) at some resolution.

With these advances, it is reasonable to anticipate that the community will amass a very large collection of chromosome interaction data in the near future. These data are expected to be collected under many different conditions as well as resolutions, and for a variety of genomes, large and small. Their processing and analysis will present tremendous challenges to bioinformaticians [32].

A number of methods have been proposed for chromosome structure inferences from the contact maps. They can either infer a mean structure from the contact map [18, 19, 33–37] or solve multiple structures [28, 38–42]. Most of these methods operate on distance matrices, which consume very large amounts of memory for genomes with high resolution.

For our method, we assume the availability of high resolution data, which gives us more information at the expense of a larger problem size. We anticipate that data will be at the level of kilobase pairs (kbp). Existing strategies have difficulties with such data. In particular, distance matrix-based methods require very large amounts of memory to work; a resolution of one kbp for the human genome would require terabytes of memory. Processing time presents another issue.

The fastest method currently has a time complexity of *O*(*n*^3^) [36], rendering them inefficient for data of large sizes. In spite of the challenge, genome-scale 3D chromatin reconstruction has nonetheless been performed in at least two studies [43, 44]. In the study by Diament el al., a sparse contact matrix was generated with only a sampled portion of the Hi-C matrix, and the reconstruction was performed with a reduced set of constraints. This method becomes inefficient when the number of loci is increased [43]. In the study by Segal et al., [44] existing single chromosome structures were incorporated to form a whole genome structure. Since the method is dependent on existing tools such as ChromSDE for the inference of single chromosome structures, they are constrained by the efficiency of those tools.

In this work, we propose a *progressive* multi-dimensional scaling (MDS) approach for structure reconstruction from Hi-C data. We introduce a succinct representation of the distance matrix to reduce space consumption. The proposed approach progressively infer the coordinates to allow more flexible control of runtime. On the benchmark dataset which consists of simulated data of 100 to 30,000 loci, our approach (implemented as a program called SuperRec) performed 5 to 435 times faster compared to ShRec3D. In particular, it demonstrated a speedup of more than 400 times in reconstructing a structure of 30,000 loci, a length sufficient for us to analyze the longest human chromosome at a resolution of 10 kbp. When accessed with normalized root-mean-square deviation (RMSD) (Additional file 1: S1), Spearman’s rank correlation coefficient (SRCC) and Pearson correlation coefficient (PCC), we found no loss of accuracy in the results obtained by SuperRec.

## Methods

This section presents our method in detail. First, we model the sequence as a continuous linear polymer. We show how the contacts from Hi-C can be transformed and represented succinctly in the form of a distance matrix. The reconstruction then works by assigning coordinates to chromosome loci progressively. After all the coordinates are assigned, we refine and sharpen the coordinates iteratively through local search.

### Structure modeling

In our algorithm, the chromosome is modelled as a continuous linear polymer, composed of many chromosomal loci. For example, human chromosome 1 can be modelled as ∼25,000 bins at a resolution (number of base pairs per bin) of 10 kbp (kilo base pairs). We use each bin to represent a locus within the chromosome. During the structure reconstruction step, the determination of each locus’s location is simplified to that of determining the 3D position of the locus’s centroid. Although this model omits the local structures at each locus, it is the most widely accepted representation and is considered the most accurate model achievable by the resolution of current 3C-based techniques [35, 36, 41]. The 3D positions of all the loci are referred to as a *structure* or a *configuration*. In this work, we let *n* denote the number of loci.

### Converting contact frequency to distance

To infer the 3D structures from Hi-C experimental data, the pairwise distances between loci are first computed. Most of the Hi-C protocols are cell population-based experiments; they provide the average contact frequencies across different cells. Every pair of loci *i* and *j* are associated with a number of *m* replicates by accumulating different structures, and the normalized contact frequency *f*_*ij*_ can be inferred from Hi-C dataset. Many structure inference methods assume a power-law relationship between contact frequency and 3D distance, allowing a contact (*f*_*ij*_) to be converted into a corresponding distance (*d*_*ij*_) through the equation $d_{ij}=1/f_{ij}^{\alpha }$. The power-law coefficient *α* varies across datasets and needs to be estimated using other techniques. The special case where *α*=1 is called an inverse frequency (IF). Given a fixed *α* and the inferred structure *X*, the goodness of fit is calculated as $\sum _{f_{ij}>0}\left (f^{\prime }_{ij}-f_{ij}\right)^{2}$ where $f_{ij}^{\prime }=1/d(X)_{ij}^{1/\alpha }$. Similar to ChromSDE, we assume that 0.1≤*α*≤3 (a range covering most *α* in previous studies) and use a golden section search to find the correct *α* through minimizing the goodness function. We face several challenges with this approach. First, Hi-C can only capture 2.5% of the contact loci, with large variations in the captured frequencies; these contacts are moreover only reliable for the physically close loci. The power-law relation $d_{ij}=1/f_{ij}^{\alpha }$ also results in infinite distances for low frequency contact pairs. While this can be remedied by enforcing an upper bound on the distances, a criteria for deciding the upper bound would be difficult to derive. Furthermore, the distances converted with a power-law relationship are not metric, rendering many computations that work for metric relations unusable.

Recently, Lesne el al. solved these problems elegantly using the shortest-path method in graph theory [36]. They modeled the contact matrix as a connected graph, in which a vertex represents a locus and an edge is associated with a distance as the inversion contact frequency of the corresponding locus pair. The final distance between loci *i* and *j* is modified with the shortest-path distance within the graph. Not only is the shortest-path distance metric and represents a tighter estimation of locus distance, the approach also mitigates the problem due to low frequency contact pairs.

Computing shortest-path distance is, however, both time- and space-consuming since it requires the computation of all-pairs shortest-paths. Hence we propose a method to approximate this distance. We randomly choose *ℓ* (*ℓ*≪*n*) loci as *pivots*, and denote this set of loci as *P*. After that, we compute the single source shortest-path distances with each pivot locus as the source to all the *n* loci. Denote the shortest distance from pivot *p* to a vertex *v* as *d*_*p*_(*v*). With these shortest-path distances from the pivots we approximate the remaining shortest-path distances. Given a pair of loci *i*, *j*, if *i* or *j* is a pivot, we can obtain their shortest distance from the computed shortest-path distances. Otherwise, we use ${\min _{p\in P}}d_{p}(i)+d_{p}(j)$ to approximate the shortest distance between *i* and *j*. By increasing the number of pivots, the approximate shortest-path distance can be made arbitrarily close to the true shortest-path distance.

To reduce the space consumption, while computing the shortest-path distances, we adopt an adjacent list representation for the distance matrix derived from Hi-C dataset. Also, the approximated distances are not stored; we merely store the *ℓ* sets of shortest-path distances from the pivots — the approximated distances are computed on the fly. This data structure reduces the space complexity from O (*n*^2^) to O(ln+e) (e is the number of significant Hi-C contacts) to store a distance matrix with *n*×*n* dimensions. See Additional file 1: Figure S8 for a comparision of run-time memory usage.

### Assigning coordinates progressively

The structure reconstruction problem is often formulated as: Given the pairwise distances (with errors) of all loci, to find a 3D configuration *X* for those loci which satisfy the distance constraints. Denote the distance between loci *i* and *j* inferred from contact information as $\hat {d}_{ij}$, and denote the Euclidean distance between loci *i* and *j* in a configuration *X* as *d*_*ij*_(*X*). The problem can be solved by minimizing the following objective function:

1 $$ \min{\sum\sum}_{i\le j\le n}\left(d_{ij}(X)-\hat{d}_{ij}\right)^{2}   $$

which can be solved by multidimensional scaling (MDS) [45]. Such an approach has been utilized by several groups to reconstruct the chromatin structures [18, 24, 36, 42]. It performs well on Euclidean distance with small error. However, the distances inferred from contact information suffer from large errors, which are especially significant in the larger distances due to the underlying mechanism of Hi-C. This prompts us to adapt the formulation to a weighted one:

2 $$ \min{\sum\sum}_{i\le j\le n}w_{ij}\left(d_{ij}(X)-\hat{d}_{ij}\right)^{2}   $$

where a weight *w*_*ij*_ can be assigned to each loci pair *i* and *j* according to their distance, *d*_*ij*_, allowing us to give higher weights to closer pairs. Similar to the earlier problem, this problem has a solution through the use of WMDS (weighted multidimensional scaling) [46].

However, the use of MDS and WMDS remains time- and space-consuming, and will not scale on problems of larger sizes. To solve this, we propose a progressive solution for the MDS and WMDS problem, namely, iMDS (iterative MDS) and sMDS (scalable MDS). Both methods relay on conducting MDS on subsets of loci.

Our proposed approaches are based on the following insight: A distance matrix of size *n*×*n* contains $n \choose 2$ variables. On the other hand, the inferred structures contain 3*n*−6 free variables. That is, the distance matrix contains information that may be considered redundant, which can be potentially discarded without affecting the quality of the inferred structure. Hence, our approach will reduce these redundant values, which we expect to lower the chances of errors. Besides this, our approach will also naturally allow the assignment of larger weights to distances that are more reliable. These will become apparent in the subsequent subsections.

#### Iterative MDS (iMDS) for structure polish

In the same way as performed in SC-MDS [47], we randomly split the set of loci into *k* overlapping subsets, *s*_1_,…,*s*_*i*_,…,*s*_*k*_, with small intersections *I*_*i*_ between *s*_*i*_ and *s*_*i*+1_. Then, classical multidimensional scaling [48] is performed on each subset to obtain the local coordinates of each locus. The subsets of loci are then combined by first selecting *s*_1_ as the reference, and then combining each *s*_*i*+1_ to *s*_*i*_ iteratively until all the subsets are combined. Our combination method differs from SC-MDS in that we incorporate the reflection of objects, which we now describe.

**Local structures recombination**

To combine *s*_*i*+1_ into *s*_*i*_ in iMDS, we use *I*_*i*_ as a set of anchors. Denote the local coordinates for *I*_*i*_ in *s*_*i*_ and *s*_*i*+1_ as *P*_*I*_ and $P^{\prime }_{I}$, respectively. We want to superimpose $P^{\prime }_{I}$ onto *P*_*I*_ with a rigid transformation (a translation *T* and a rotation *R*) such as to minimize the RMSD. This problem is known to be solvable in linear time [49]. In order to solve the reflection problem, $P^{\prime }_{I}$ and its mirror were both superimposed to *P*_*I*_. In the case that the RMSD between the mirror of $P^{\prime }_{I}$ and *P*_*I*_ is smaller than that between $P^{\prime }_{I}$ and *P*_*I*_, the coordinates in *s*_*i*+1_ are replaced with those in the mirror of *s*_*i*+1_.

3 $$ RMSD=\min\sum\limits_{i=1}^{N}\sqrt{\left\| p_{i}^{\prime}-(Rp_{i}+T)\right\|^{2}}   $$

After obtaining *T* and *R*, we apply them to the coordinates of *s*_*i*+1_ such that *s*_*i*+1_ and *s*_*i*_ would have the same frame of reference. This integrates *s*_*i*+1_ into *s*_*i*_. In addition, we average *P*_*I*_ and the transformed coordinates of $P^{\prime }_{I}$ by *R* and *T* to update the coordinates of *I*_*i*_.

**Successive subsetting and combination** As discussed in [47], grouping only neighboring loci is not as beneficial as grouping both close and distant loci. Hence, we randomly split all loci into *k* overlapping subsets. In order to combine two 3D sets successfully, we need to select at least 4 points not lie on the same plane (points on the same plane cannot identity a structure in 3D space), additionaly, a small number of loci may lead to a poor estimation of the rotation and translation matrix. Hence, we need to choose enough loci in *I*_*i*_. In practice, we set the number of loci in *I*_*i*_ as 50, which is large enough for successive combinations in a three dimensional space. The performance of random subsetting and intersection is described in Additional file 1: S2.

Due to Hi-C’s mechanism, the distances $\hat {d}$ measured have different degrees of reliability, which may aversely affect MDS and iMDS. This situation worsens when noise is elevated. To address this, we propose a scalable MDS to polish the structure obtained by iMDS.

#### Incorporating different distances

The structure from iMDS is further improved to better agree with the data from Hi-C experiments. In Eq. 2, a weight *w*_*ij*_ was introduced for each distance $\hat {d}_{ij}$ from Hi-C. Since shorter distances are more reliable than the longer ones, we set $w_{ij}=\hat {d}_{ij}^{-2}$ [42] to decrease the influence from the longer distances. We found this weighting scheme to be more robust than other schemes such as *w*_*ij*_=1 and $w_{ij}=1/\hat {d}_{ij}$ [35, 36] (Additional file 1: S3).

However, computing WMDS for our framework remains infeasible for large data sets due to time and space complexities. We propose a scalable MDS approach here to address this issue. As far as we know, our approach is among the very few that are currently available for large-scale WMDS.

#### Scalable MDS

Our proposed scalable MDS is an iterative procedure which employs WMDS as a subroutine. At each iteration, we permute the loci randomly, and partition them by the permuted order into sets of size *k* each. Then, we apply WMDS to each resultant set, *S* say. Denote the loci coordinates in *S* before executing WMDS and after as *P*_*S*_ and $P_{S}^{\prime }$ respectively. We apply RMSD to discover the optimal superposition that maps each locus in $P_{S}^{\prime }$ to itself in *P*_*S*_. Then, we update the coordinates for the locus to its average values from *P*_*S*_ and $P_{S}^{\prime }$ after the superposition. After we iterate through every set *S*, we restart the process for another round. This is repeated until the coordinates of the loci converge. The performance of random subsetting and iteration is described in Additional file 1: S2 and the sensitivity analysis of parameter settings is described in Additional file 1: S4.

WMDS is an iterative algorithm, the time complexity in each iteration is O (*n*^3^). In comparsion, the time complexity of sMDS for updating all loci once is O (*nk*^2^). Though sMDS need more iterations than WMDS (10 times more in practice), sMDS is faster.

### Simulated contact maps

#### Binary contact map

We used known 3D structures as the basis for our simulation. For a given 3D structure, we constructed a binary matrix to store the pairwise contact information, with the top *k* nearest pairs of loci set to 1 and others set to 0.

#### Poisson distribution model

For a given 3D structure with pairwise distance *d*_*ij*_, We generated the *M*(*i,j*) entry of a simulated contact map *M* as a Poisson-distributed random number *n*_*ij*_. The parameter *λ*_*ij*_ is defined as $\lambda _{ij}=\beta /d_{ij}^{\alpha }$ based on the power-law conversion of distance and contact frequency. *β* is a tunable parameter to control the signal coverage of the contact map.

### Data preparation

#### In silico genome structure

The in silico nucleus with 1 and 16 chromosome(s) (Additional file 1: Table S1) used to test our software were generated using a polymer model. The coordinates were taken from the Langevin dynamics simulation after it has reached thermal equilibrium. After that, we constructed Poisson distribution-based contact maps and binary contact maps for the in silico structures. In addition to the contact matrices corresponding to in silico chromosomes, we also included contact maps generated from Poisson distribution model at different signal coverage levels of the regular helix structure used as benchmark dataset by Zou et al. [37] as well as the structure of chromosome 2 reconstructed from a real Hi-C dataset (mESC).

#### Hi-C experimental dataset

In this study, we used Hi-C data from two cell lines mESC and GM12878. These raw sequence data are transformed into contact map with Juicer [50]. The matrix balancing method described in [51] was used to normalize the contact matrix in order to remove biases in Hi-C dataset.

## Results and discussion

We implemented our proposed approach in a package named *SuperRec*. We compared SuperRec with public publicly available softwares on a ubuntu 16.04 server equipped with two Intel(R) Xeon(R) E5-2620 CPUs, and 256 GB memory. All softwares were executed with suggested configurations, default setting were used when there is no recommended configuration.

### The approximated shortest-path distances are reliable

**Fig. 1:**
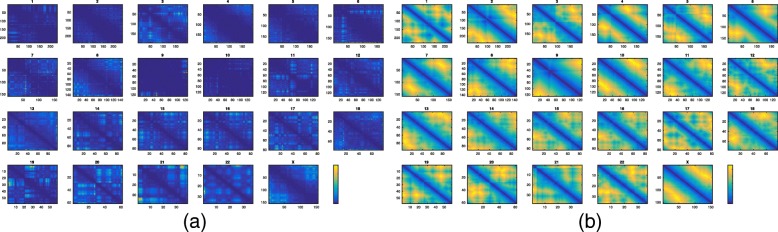
Pairwise distances within each chromosome: **a** distances converted with power-law conversion, **b** power-law converted distances with the refinement of all-pair shortest-path distance

We first assessed the quality of our approximation of the shortest-path distance. Figure 1 visualizes the Hi-C dataset SRX764938 (GM12878) [52] of human. Initially, there are many distances of small values derived from power-law conversion that are indistinguishable from each other (Fig. 1a). After using the shortest-path distances to refine the power-law converted distances, the local distances along the genome became significantly closer than the longer-range ones as expected (Fig. 1b).

An effective solution here is to use the shortest-path distance refinement approach to infer the spatial distances. However, the approach is time- and space-consuming, requiring time cubic to the number of loci; the very large number of loci to compute for renders it infeasible.

To overcome this we approximated the shortest-path distances through the use of pivots as described earlier. To assess the effects of the accuracy loss, we attempted to reconstruct the 3D configuration from the approximated distances of an in silico dataset with 10,000 loci. We repeated this for a range of different number of pivots from 1 to 10,000 to examine how having more pivots would affect the result; the case of using 10,000 pivots is the same as when the actual shortest-path distances are used.

**Fig. 2:**
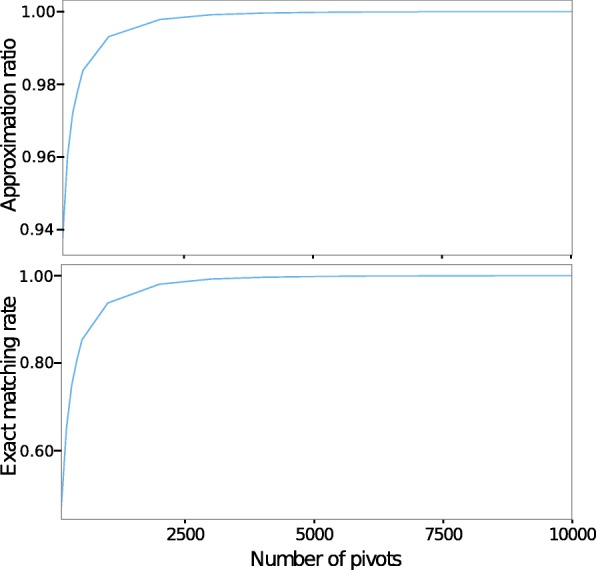
Accuracy of approximate shortest-path distance: Approimation ratio calculated as the ratio of sum of all-pair shortest-path distance; Number of matched pairs of distance between approximate shortest-path distance and real shortest-path distance

We first assessed the accuracy loss due to approximation through two parameters: *Approximation Ratio (AR)* and *Exact Matching Rate (EMR)*. The approximation ratio is defined to be the ratio between the actual all-pair shortest-path length and the approximated all-pair shortest-path length. We obtained favorable AR values of 0.93 for 100 pivots, and 0.98 for 500 pivots. With 1000 pivots, the approximation ratio became more than 0.99 (Fig. 2a). The exact matching rate is defined to be the rate of the approximated shortest paths lengths that are equal to the corresponding actual shortest-path length. We obtained EMR of 0.85, 0.93 and 0.98 with 500, 1000, and 20,00 pivots, respectively (Fig. 2b). Additionaly, both EMR and AR show very small variances in our repeated analysis (Additional file 1: Figure S9).

**Fig. 3:**
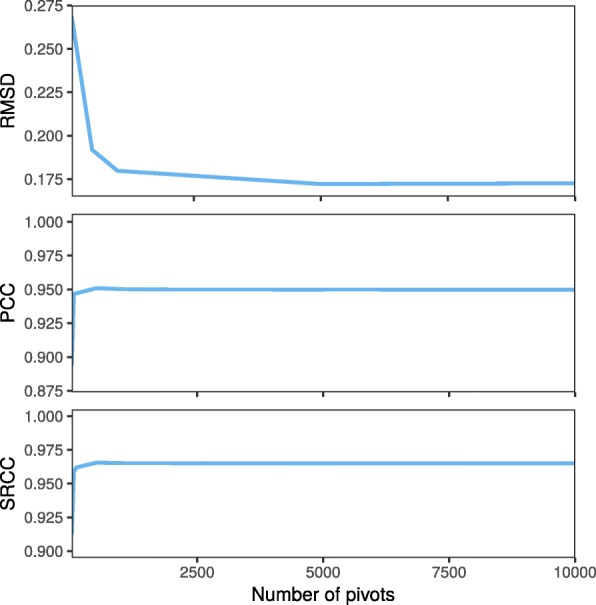
Accuracy analysis of a reconstructed structure using various numbers of pivots to compute the shortest-path distance. For cases with more than 500 pivots, the difference between structures from accurate shortest-path distances and those from approximated shortest-path distances is negligible. Normalized RMSD, PCC as well as SRCC measurements are consistent

We next examined if the quality of our reconstruction would be affected when the approximated shortest-path distances are used instead of the actual shortest-path distances. We conducted experiments on the same data with 10,000 loci, and with varying number of pivots from 1 to 10,000. The PCC between pairwise distance calculated from reconstructed and true structures was found to be 0.89 when 50 pivots are used. These values converge to 0.95 when more than 100 pivots are used. Similarly, the SRCC between pairwise distances calculated from reconstructed and real structures converge to 0.965 when more than 100 pivots are used. The normalized RMSD between reconstructed and real structure varies from 0.17 to 0.27 throughout the experiment (Fig. 3); these values are considered small since they are the aggregate of 10,000 loci. These show that there is no significant degradation of result from the use of the approximated shortest-path distances. In our experiment, SuperRec performs well when using 10% or more loci as pivots (Additional file 1: Figures S6, S7), and we suggest using at least 10% loci as pivots when using SuperRec.

### SuperRec is fast

**Fig. 4:**
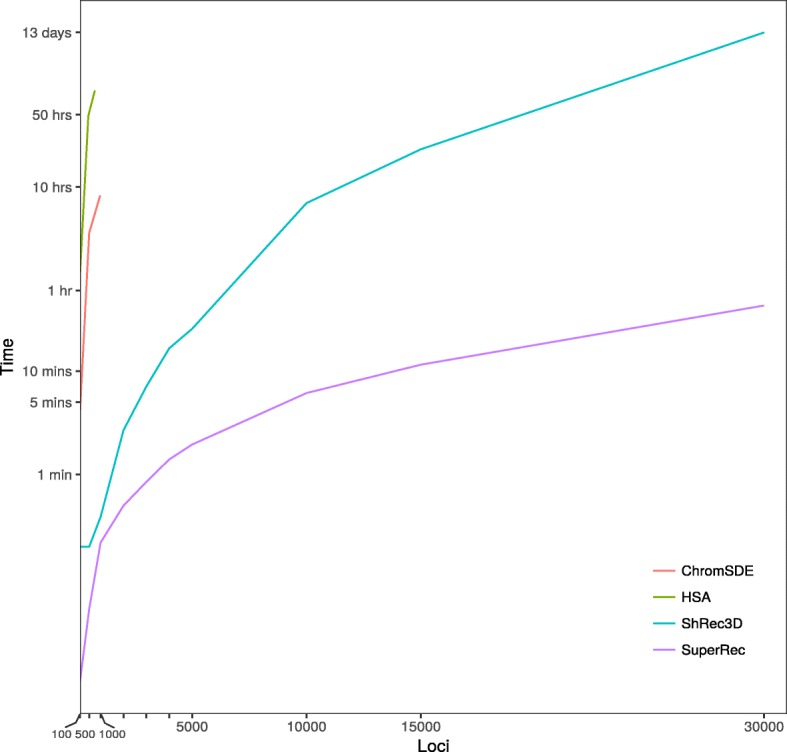
Time usage for reconstructing chromosomal structures

To compare the speed of SuperRec with existing methods, we simulated chromosomal structures with different numbers of loci up to 30,000 (Additional file 1: Table S1). The corresponding binary contact information were inferred with in silico structures as described in Method. These binary contact data were further analyzed with SuperRec using 10% loci as pivots as well as ChromSDE, HSA [37], and ShRec3D. Figure 4 shows the computation time plotted against the number of loci. SuperRec achieves significant improvements when handling thousands of loci. For large dataset with 2000 loci and more, SuperRec performed between 5 to 435 times faster than its alternatives. In one instance with 30,000 loci, SuperRec took only 43 min, whereas ShRec3D required more than 13 days. ChromSDE and HSA would require a few hours to complete on cases with more than 1000 loci Hence, we stopped the comparison with ChromSDE and HSA on the larger cases.

### SuperRec is accurate

**Fig. 5:**
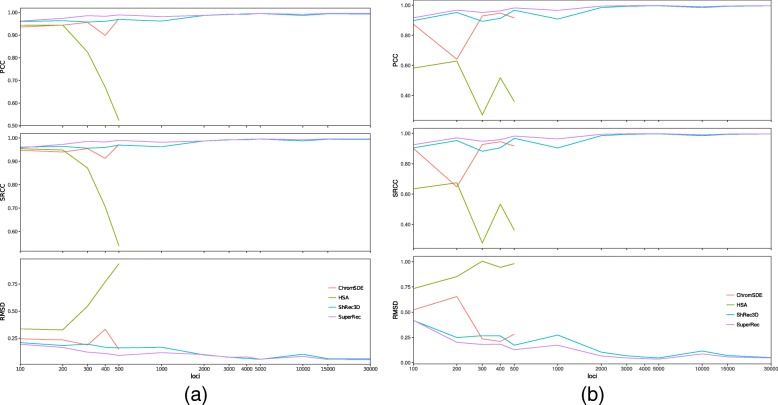
Accuracy measurement by normalized RMSD, PCC and SRCC vs different numbers of loci. For structures with large number of loci, the SRCC and PCC is almost one, which indicated the reconstructed structures are close to the original structures. We stopped ChromSDE, and HSA computation beyond 500 loci due to the high runtime: **a** Poisson distribution-based contact maps; **b** Binary contact maps

Whereas we have used only synthetic data in our speed benchmark test, the quality assessment of our algorithm was performed using both synthetic data and actual Hi-C experimental data. We first performed the reconstruction from the synthetic contact matrix used (Additional file 1: Table S1) using HSA, ChromSDE, ShRec3D, and SuperRec. To achieve a fair and comprehensive comparison, three different measurements were calculated: (1) normalized RMSD of each pair of reconstructed structure and original structure, (2) PCC and (3) SRCC between the original and the reconstructed distances (Fig. 5). On Poisson distribution-based contact map datasets, all algorithms achieved comparable and accurate results under all three kinds of measurement, consistently reporting correlation coefficients greater than 0.90. The corresponding normalized RMSDs reported are small compared to the number of loci, except for HSA with 400 and 500 loci. On binary contact maps datasets, SuperRec and ShRec3D achieved comparable and accurate results under all three kinds of measurement, consistently reporting correlation coefficients greater than 0.90. The corresponding normalized RMSDs reported are also small compared to the number of loci. In contrast, the performance of HSA and ChromSDE is dissatisfactory due to their inability to handle binary contact map. This shows that SuperRec is as accurate as these state-of-the-art algorithms.

**Fig. 6:**
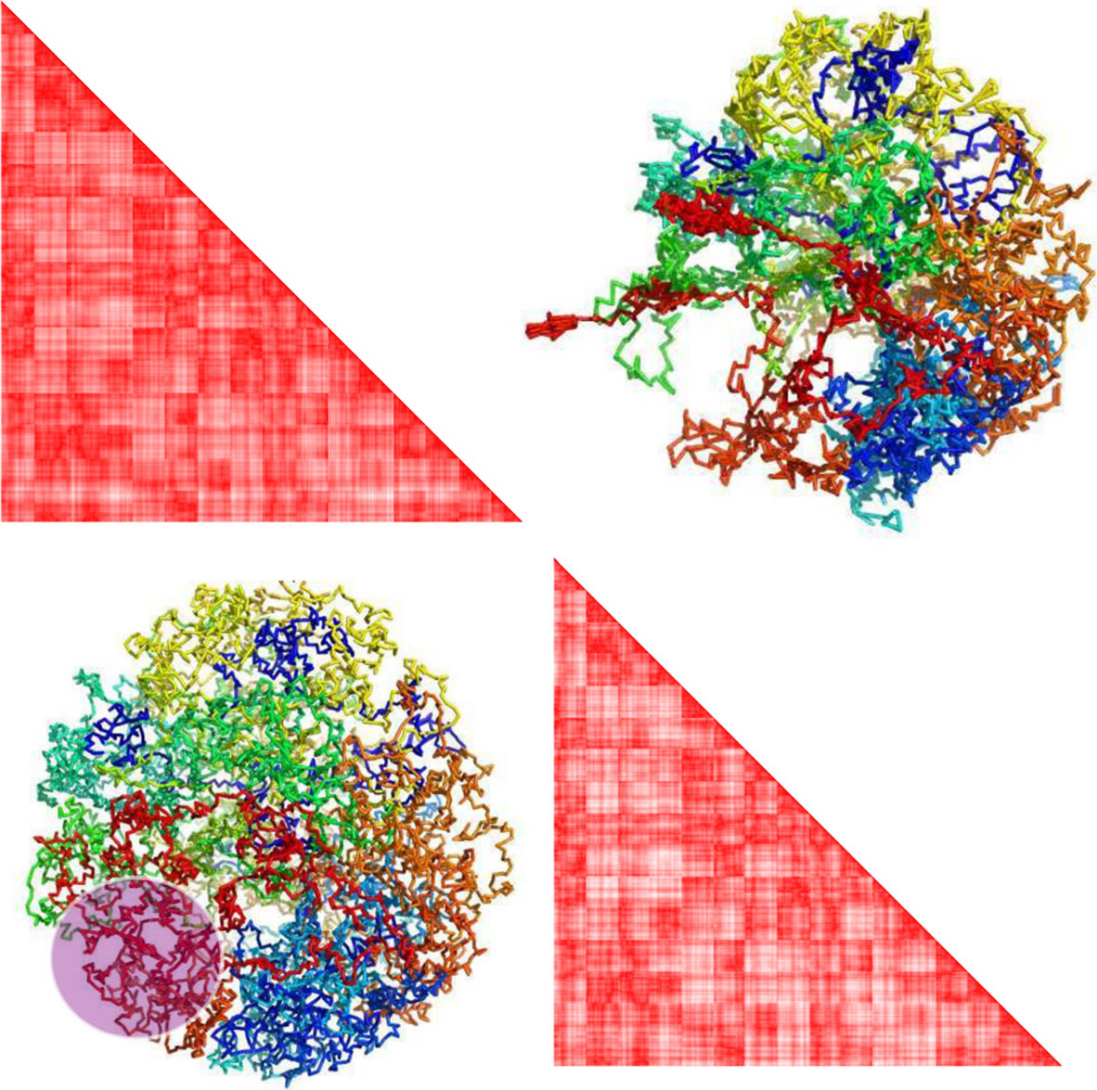
The upper right and bottom left are two structures with 16 chromosomes and 10,000 loci: the upper right corresponding to a reconstructed structure by SuperRec with 1000 pivots, while the bottom left is the corresponding original structure. The upper left and bottom right heatmaps are plotted using pairwise distances inferred from reconstructed and real structures respectively

**Fig. 7:**
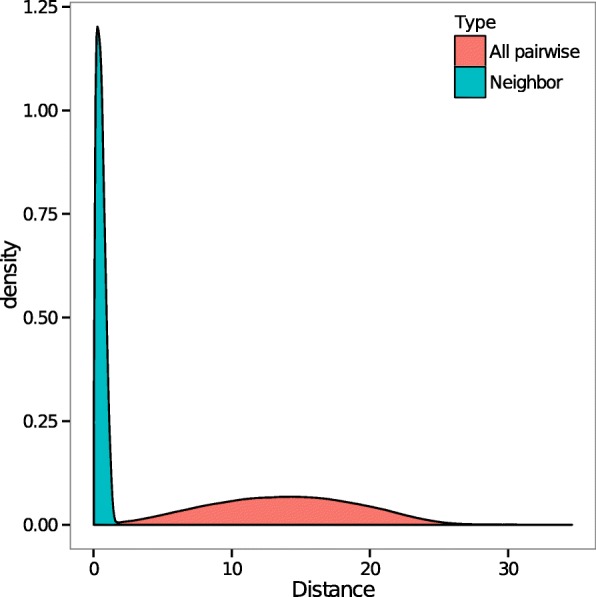
Polymer connectivity: Histogram of distances between neighboring loci along chromosomal sequence (blue) and all pairwise distances (red) computed from a reconstructed configuration of 10,000 loci

Figure 6 shows two structure: an in silico structure with 10,000 loci, and the structure reconstructed by SuperRec. The two structures are highly similar except for the purple highlighted region. The discrepancy is likely due to the relative sparsity of loci in the highlighted region, which resulted in the loci of that region to have fewer contacts with others, thus complicating the inference. On the other hand, we note that the polymer connectivity is preserved in our reconstructed structure (Fig. 7).

In addition to the comparison based on synthetic chromatin structures, we also reconstructed the structure of the regular helix from contact matrices at different signal coverage levels with HSA, ShRec3D, ChromSDE as well as SuperRec. Evaluating by the normalized RMSD between reconstructed structure and real structure, we find our method to be effective and robust (Additional file 1: Table S2).

**Fig. 8:**
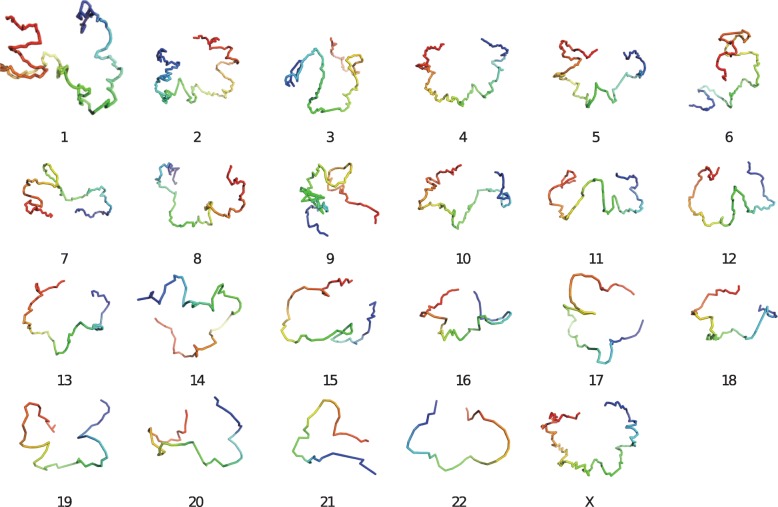
3D visualization of human autosomes and X chromosome based on Hi-C data (SRX764938) [52] at 1MB resolution, only intra-contact frequency account for those reconstructions

We also performed the analysis with an *in situ* Hi-C dataset (GM12878) [52] of human at 1MB resolution. The chromosome (Fig. 8) reconstructions were performed with the usage of intra-contact matrices. Since the underlying structure of the Hi-C dataset is unknown, to evaluate the accuracy of the reconstructed structures we compared the pairwise distances from the reconstructions with the all pair shortest-path distances calculated from contact frequency. The similarity is then expressed using correlation coefficients (Table 1). ShRec3D and SuperRec achieved similar performances when handling chromosome level reconstruction. At genome-wide level of number of loci, SuperRec slightly outperformed ShRec3D.

**Table 1:** Accuracy measurement of SuperRec, ShRec3D and HSA based on real Hi-C data using Spearman’s rank/Pearson’s coefficient between distances calculated from reconstructions and all-pair shortest-path distances computed from contact information

Correlation	Method	Chromosome																							
		1	2	3	4	5	6	7	8	9	10	11	12	13	14	15	16	17	18	19	20	21	22	X	All
Spearman	SuperRec	0.981	0.986	0.973	0.942	0.971	0.964	0.989	0.993	0.997	0.981	0.987	0.979	0.991	0.992	0.976	0.976	0.973	0.980	0.979	0.974	0.974	0.970	0.982	0.880
	ShRec3D	0.981	0.983	0.984	0.988	0.985	0.989	0.990	0.987	0.998	0.991	0.977	0.981	0.985	0.991	0.986	0.986	0.974	0.983	0.986	0.982	0.992	0.979	0.990	0.851
	HSA	0.799	0.916	0.916	0.740	0.857	0.884	0.866	0.952	0.904	0.904	0.892	0.801	0.733	0.891	0.880	0.873	0.759	0.808	0.747	0.841	0.934	0.828	0.892	-
Pearson	SuperRec	0.982	0.985	0.974	0.947	0.974	0.960	0.987	0.993	0.998	0.982	0.989	0.983	0.989	0.992	0.980	0.980	0.978	0.985	0.982	0.972	0.970	0.978	0.981	0.896
	ShRec3D	0.981	0.978	0.983	0.976	0.977	0.985	0.986	0.988	0.998	0.983	0.983	0.980	0.984	0.990	0.984	0.984	0.974	0.984	0.985	0.980	0.991	0.977	0.979	0.857
	HSA	0.820	0.915	0.904	0.719	0.812	0.831	0.835	0.935	0.909	0.897	0.879	0.813	0.757	0.893	0.851	0.890	0.790	0.813	0.752	0.786	0.914	0.763	0.836	-

### Validations and comparisons using FISH data

**Fig. 9:**
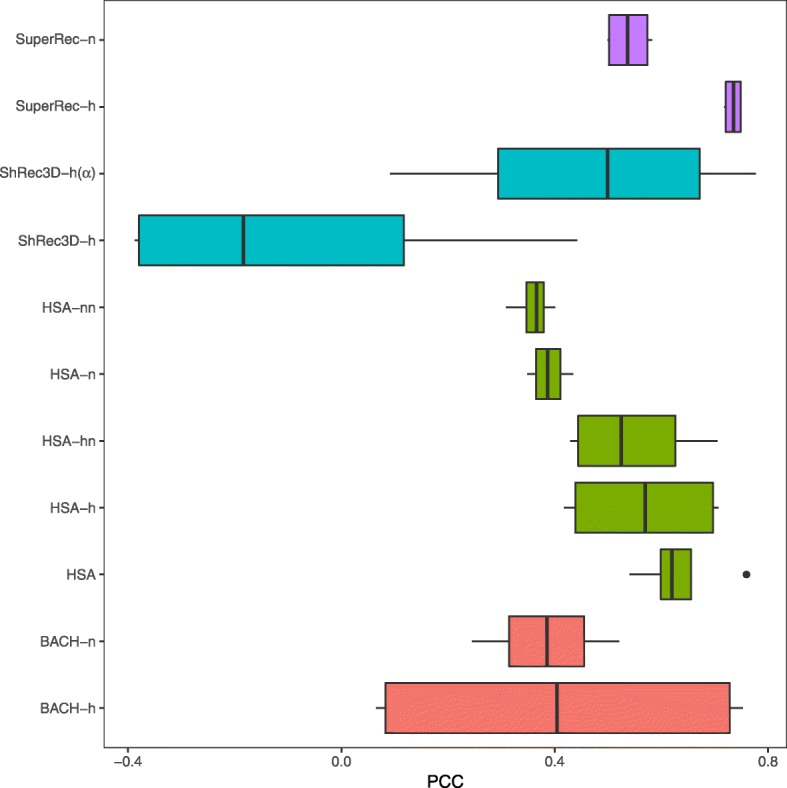
Box plots of PCCs between FISH measured distance and predicted distances by different Hi-C dataset of mESC at 40-kb resolution. *BACH-h* BACH with contact map of HindIII, *BACH-n* BACH with contact map of NcoI, *HSA* multi-track HSA, *HSA-h* HSA with contact map of HindIII, *HSA-nn* HSA with normalized contact map of NcoI, *HSA-hn* HSA with normalized contact map of HindIII, *HSA-n* HSA with contact map of NcoI, *ShRec3D-h* ShRec3D with normalized contact map of HindIII, *ShRec3D-h(α)* modified ShRec3D with given *α* on normalized contact map of HindIII, *SuperRec-h* SuperRec with normalized contact map of HindIII, and *SuperRec-n* SuperRec with normalized contact map of NcoI

We also compared the methods’ performance in inferring 3D chromatin structures using known distances derived from public 3D-FISH data for the cell lines mESC [53]. We selected six pairs of genomic loci from chromosome 2 or chromosome 11 for validation, with distances derived from FISH probes at 40-kb resolution. We inferred structures of chromosome 2 and chromosome 11 with HSA, BACH, ShRec3D and SuperRec with Hi-C contact data at 40-kb resolution. Single-track HSA, multi-track HSA and BACH were executed with the raw contact maps of NcoI and HindIII. ShRec3D and SuperRec were executed with the normalized contact maps of NcoI and HindIII. In addition, a modifed version of ShRec3D with a fixed *α* for distance conversion was included in our analysis. PCCs between the predicted distances from reconstructed structures and distance from 3D-FISH were calculated. Since each FISH locus spans two neighboring loci in the structures derived from Hi-C dataset, different combinations of neighbor loci at the two ends of FISH probed pair were used to compute the distances from 3D structures, and a range of PCCs for each FISH data set were obtained. The PCCs of SuperRec and multi-track HSA are most robust and significantly higher than those of other approaches (Fig. 9).

### Application to sparse contact map

**Fig. 10:**
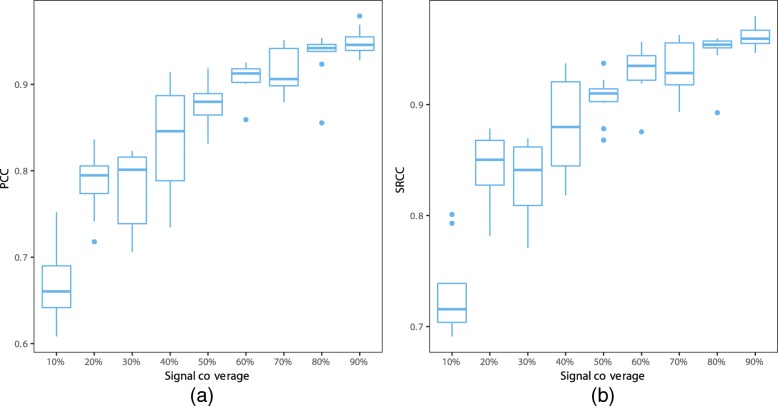
Box plots of PCC (**a**) and SRCC (**b**) between true distances and predicted distances by simulated contact maps at different signal coverage

We carried out reconstructions using SuperRec on simulated contact maps of chromosome 2 (mouse) at different signal coverages (from 10 to 90%), and 10 simulated contact maps were generated at each signal coverage. SuperRec works well for both sparse and dense contact map when accessed with PCC and SRCC between distances from reconstructed structure and true structure. PCC ranges between 0.60 and 0.75, SRCC ranges between 0.65 and 0.80 at 10% signal coverage. The correlations increased with increasingly high signal coverage, with both PCC and SRCC approaching 0.9 when signal coverage reaches above 50 (Fig. 10). This demonstrates SuperRec’s ability in handling Hi-C contact maps from low to high coverage.

## Conclusion

In this study, we devised a novel method for 3D chromatin reconstruction from chromosomal contacts, and implemented it into a complete software solution called *SuperRec*. We tested SuperRec on both synthetic and real Hi-C datasets. SuperRec achieved significant improvements in the analysis of longest human chromosome, completing the reconstruction at a resolution of 10 kbp within hours without loss of accuracy in the results.

## Additional file

Additional file 1 Large Scale 3D Chromatin Reconstruction From Chromosomal Contacts - Supplementary materials. This file contains the supplementary text and figures mentioned in the text. (PDF 6926 kb)

## Abbreviations

3C: Chromosome conformation capture
AR: Approximation ratio
EMR: Exact matching rate
FISH: Fluorescence in situ hybridization
IF: Inverse frequency
iMDS: Iterative multi-dimensional scaling
kbp: Kilobase pairs
MDS: Multi-dimensional scaling
PCC: Pearson correlation coefficient
RMSD: Root mean square deviation
sMDS: Scalable multi-dimensional scaling
SRCC: Spearman’s rank correlation coefficient
WMDS: Weighted multi-dimensional scaling
