# Supplementary material for: Large-scale 3D chromatin reconstruction from chromosomal contacts
Source: BMC Genomics. 2019 Apr 4;20(Suppl 2):186. doi: 10.1186/s12864-019-5470-2 (PMC6456969; doi:10.1186/s12864-019-5470-2)
Supplement: Supplementary file 1 — Large Scale 3D Chromatin Reconstruction From Chromosomal Contacts - Supplementary materials. This file contains the supplementary text and figures mentioned in the text. (PDF 6926 kb) [file 12864_2019_5470_MOESM1_ESM.pdf]

Large Scale 3D Chromatin Reconstruction From Chromosomal Contacts - Supplementary materials

Yanlin Zhang, Weiwei Liu, Yu Lin, Yen Kaow Ng, and Shuai Cheng Li

S1 Normalized RMSD

Structures inferred from different data sets or with different methods may suffer from scale differences. In order to compare these structures, we use a *normalized* RMSD [1].

For a given 3D configuration X with n points, we denote the mean of the points in X as $\bar{X}$. We compute a scale factor s from $\bar{X}$ as:

$$s = \sqrt{\frac{1}{n}\sum_{i=1}^{n}[(x_{i1} - \bar{X}_1)^2 + (x_{i2} - \bar{X}_2)^2 + (x_{i3} - \bar{X}_3)^2]} \quad (1)$$

The uniform scaling structure of X can be obtained as X/s.

For two given 3D structures p and p', we first remove the scale by converting them to uniform structures, then use RMSD to measure the structure similarity:

$$\text{RMSD} = \min \sum_{i=1}^{N} \sqrt{\|p'_i - (Rp_i + T)\|^2} \quad (2)$$

where R is a 3×3 rotation matrix, and T is a 3×1 translation vector.

S2 Subsetting and combination

iMDS

Subsetting To access the performance of subsetting, we set the number of overlapped loci to 50, which is a reliable value to combine overlapped structures. We executed iMDS on a structure of $10,000$ loci (same as the one in our main text) with different sizes of subsets (i.e. 100, 200, 300, 400, 500, 600, 700, 800, 900, 1000, 2000, 3000, 4000, 5000) using their exact shortest-path distances. Besides that, we also performed another series of iMDS by grouping spatially close loci into the same set. Then we performed classical multidimensional scaling (CMDS) on all the $10,000$ loci. To analyze the effect of subsetting, we compared the structures generated with and without subsetting using normlized RMSD (Figure S1). We found that when the number of loci in a set is increased, the normlized RMSD tends to be smaller with both subsetting methods, and that iMDS with random subsetting better approximates CMDS than iMDS with grouping close loci. Furthermore, RMDS tends to be 0 when the number of loci in a set is around $1,000$ or more.

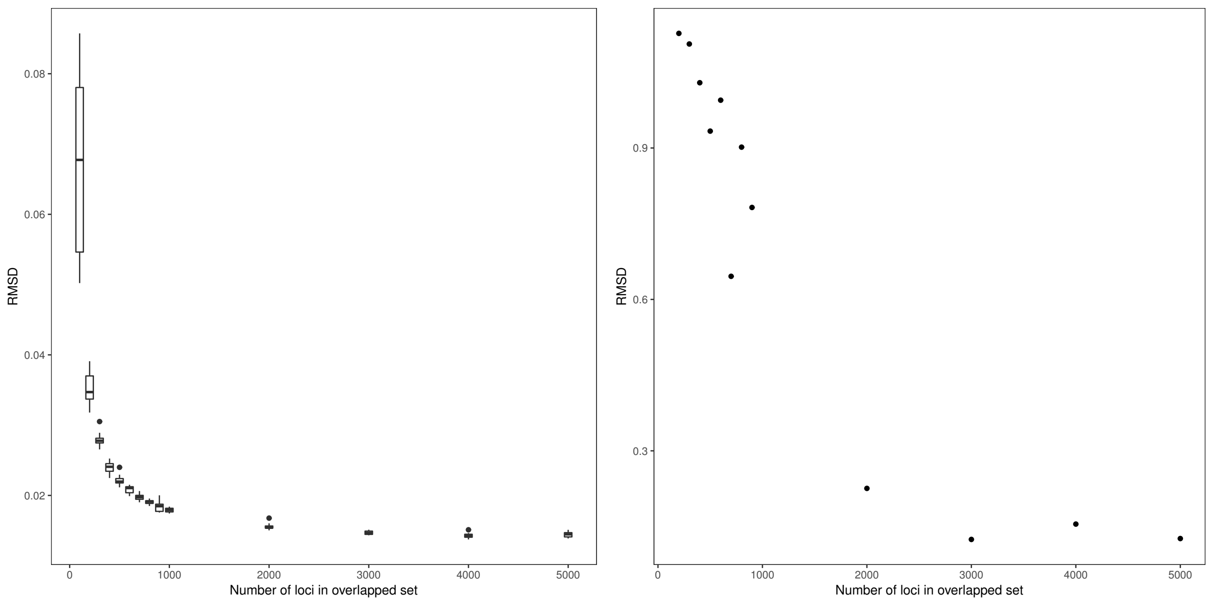

(a) (b)

Figure S1: Normalized RMSD calculated between coarse-grain structures inferred by iMDS with different group size and structure inferred by classical multidimensional scaling without splitting. (a) Loci were randomly split into different sets. 10 replicates were performed with each group size. (b) Loci were grouped into sets such that close loci formed a set.

Overlapped loci We generated an *in silico* 3D chromosome with $2,000$ loci, as well as its corresponding contact matrix, following the procedure in the main text. To be able to combine two substructures in three dimensional space, the number of overlapped loci is to be at least 3. Hence, we randomly split $2,000$ into two overlapped sets with $1,000 + r$ loci in each set, where r is the number of overlapped loci. We performed iMDS on both datasets using the exact shortest-path distances with different number of overlapped loci (3, 4, 5, 6, 7, 8, 9, 10, 20, 30, 40, 50, 60, 70, 80). We also performed CMDS on all $2,000$ loci without subsetting. To analyze how overlapped loci affect combination, we compared the structures generated with and without subsetting (Figure S2). Two sub-structures ($\sim$1,000 loci in each) can be combined successfully when there are more than 3 overlapped loci. Hence, iMDS is a reliable approximation of classical multidimensional scaling with more than 3 overlapped loci with a group size of $\sim$1,000.

Scalable MDS

Subsetting To test the performance of our scalable MDS, we executed scalable MDS with different set sizes (100, 200, 300, 400, 500, 600, 700, 800, 900, 1000, 2000, 3000, 4000, 5000) on the same structure we used in subsetting sections of iMDS. To minimize the influence of iMDS, we used structures inferred from classical multidimensional scaling on all $10,000$ loci as the initial structure of scalable MDS. Since we do not have weighted MDS program that can handle $10,000$ loci, we compared structures inferred by scalable MDS with the true structure using normlized RMSD (Figure S3). The difference between the structures reconstructed with scalable MDS and the true structure is negligible when the set size is greater than $1,000$.

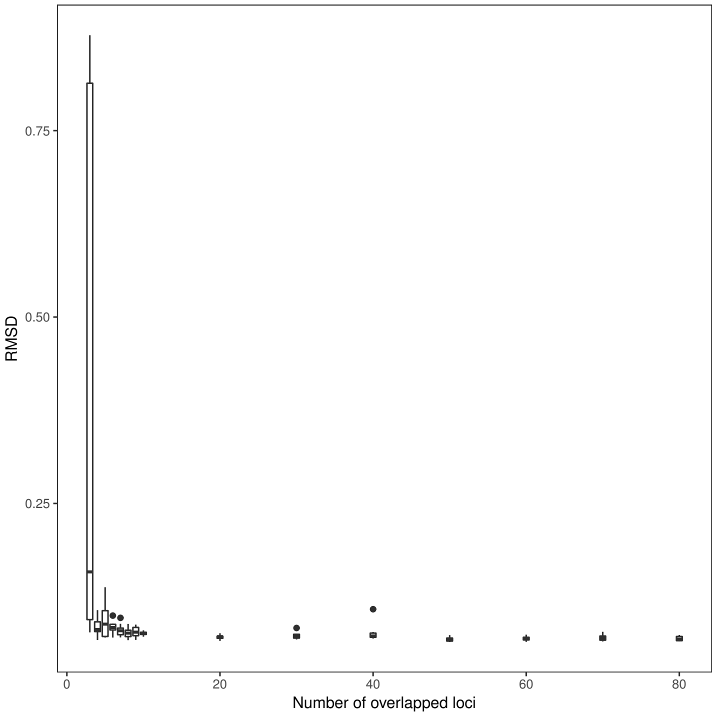

Figure S2: Normalized RMSD calculated between coarse-grain structure inferred by iMDS with a group of 1000 loci with a different number of overlapped loci and structure inferred by classical multidimensional scaling without splitting.

Iteration To study the behaviour of iteration in Scalable MDS, we compared the normalized RMSD between reconstructions and in silico structures of different loci (2,000, 3,000, 4,000, 5,000, 15,000, 30,000). For any in silico dataset, 10 scalable MDS replicates were performed, and we provided scalable MDS with the same initial structure and approximate shortest path distance in each replicate. Based on our experiments, the normalized RMSD decreased with the increasing of iterations (Figure S4).

S3 Weight schemes

To compare three different weight schemes, $w = \frac{1}{d}$, $w = \frac{1}{d^2}$, and $w = 1$, we generated an *in silico* structure with 450 loci, as well as 10 different contact maps associated with the *in silico* structure at different signal coverages and noise levels. To make a fair comparison, we used exact shortest-path distances instead of the approximated shortest-path distances, and we also set the size of each set to 450 to disable subsetting. Pearson correlation were calculated between the *in silico* structures and the reconstructed ones (Figure S5).

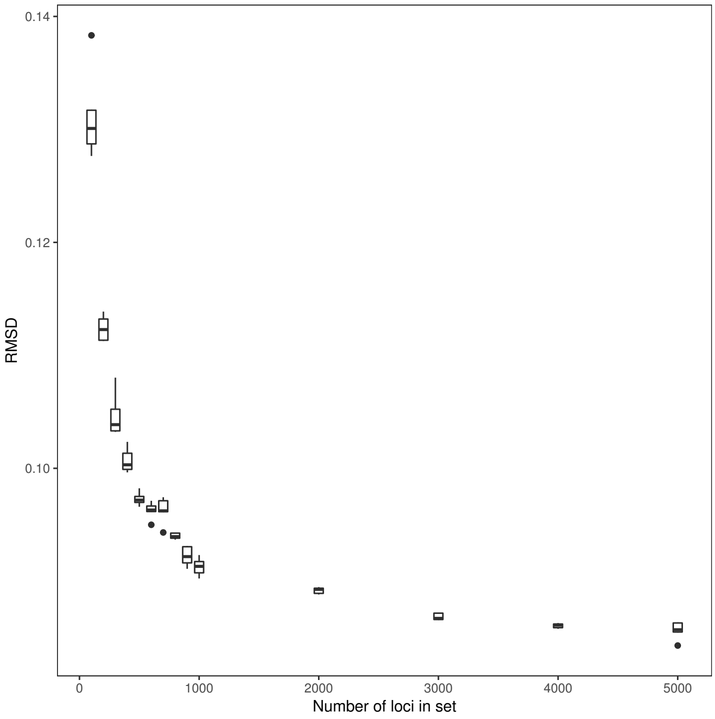

Figure S3: Normalized RMSD calculated between structure inferred by scalable MDS and real structure. Loci were randomly split into different sets. 10 replicates were performed with each group size.

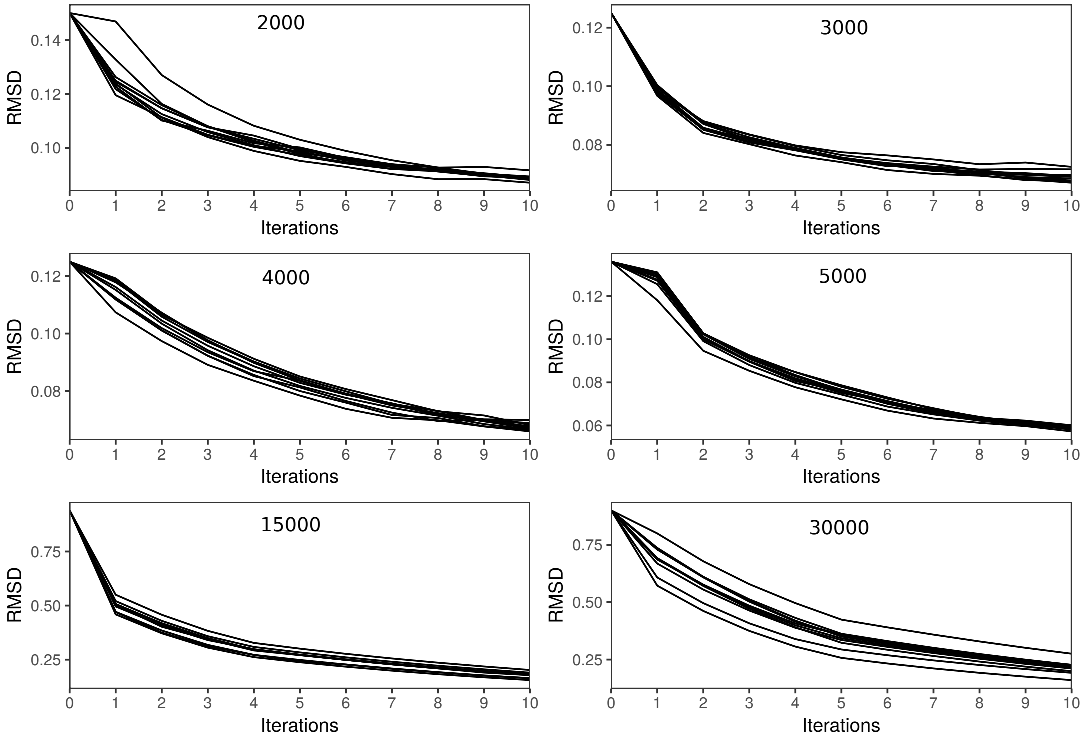

Figure S4: Normalized RMSD calculated between real structures and structures inferred by scalable MDS with different number of iterations. 10 replicates were performed with each dataset.

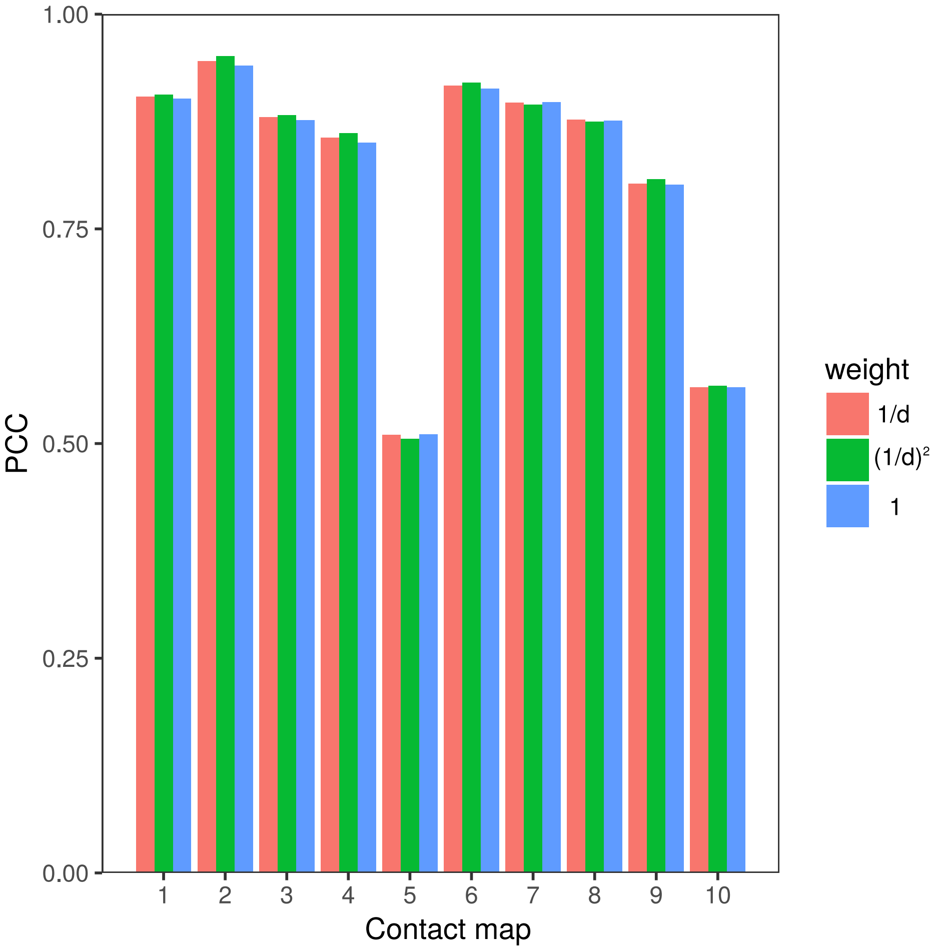

Figure S5: Comparison between different weight schemes ($w = \frac{1}{d}$, $w = \frac{1}{d^2}$ and $w = 1$) based on PCC between distances calculated from *in silico* strucutures and from reconstructed structures.

S4 Combination of different parameters in SuperRec

To anslysis the sensitivity of paramters in SuperRec. We conducted experiments with different combinations of parameters (pivots, overlaps, set size). We found when grouping 400 or more loci in a set, no matter how we set other parameters, SuperRec can produce similar results after 10 sMDS iterations. For small clusters, more sMDS iterations are required. Our default settings are the recommendations, and in most cases, users do not need to change them. The sensitivity anslysis was conducted with two *in silico* structures with 2,000 and 10,000 loci, a structure of this scope is large enough for most 3D genome analysis tasks (Figure S6 and S7).

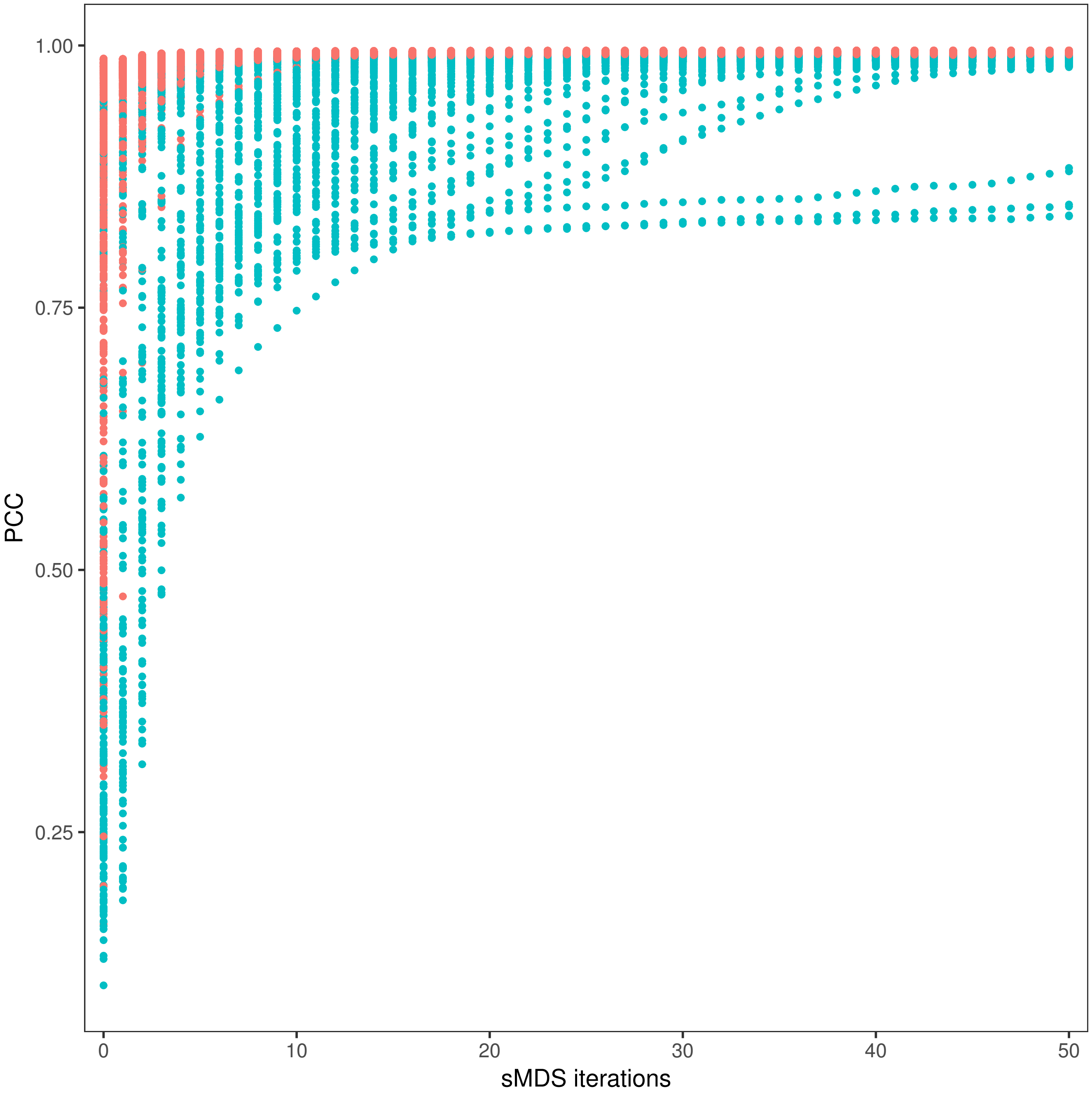

Figure S6: Comparison of reconstructed structures calculated with a different number of sMDS iterations and *in silico* structure. A combinations of different parameter settings (pivots={100,200,300,400,500,1000,2000}, overlaps={3, 4,. . . ,9, 10, 20,. . . ,80, 90,100}, and set size={100,200,. . . ,900,1000,2000}) were used, each dot on the plot corresponding to one set of parameters. Red dots corresponding to experiments with set size less than or equal to 200, and blue dots corresponding to experiments with set size larger than 200. The *in silico* structure contains 2,000 loci.

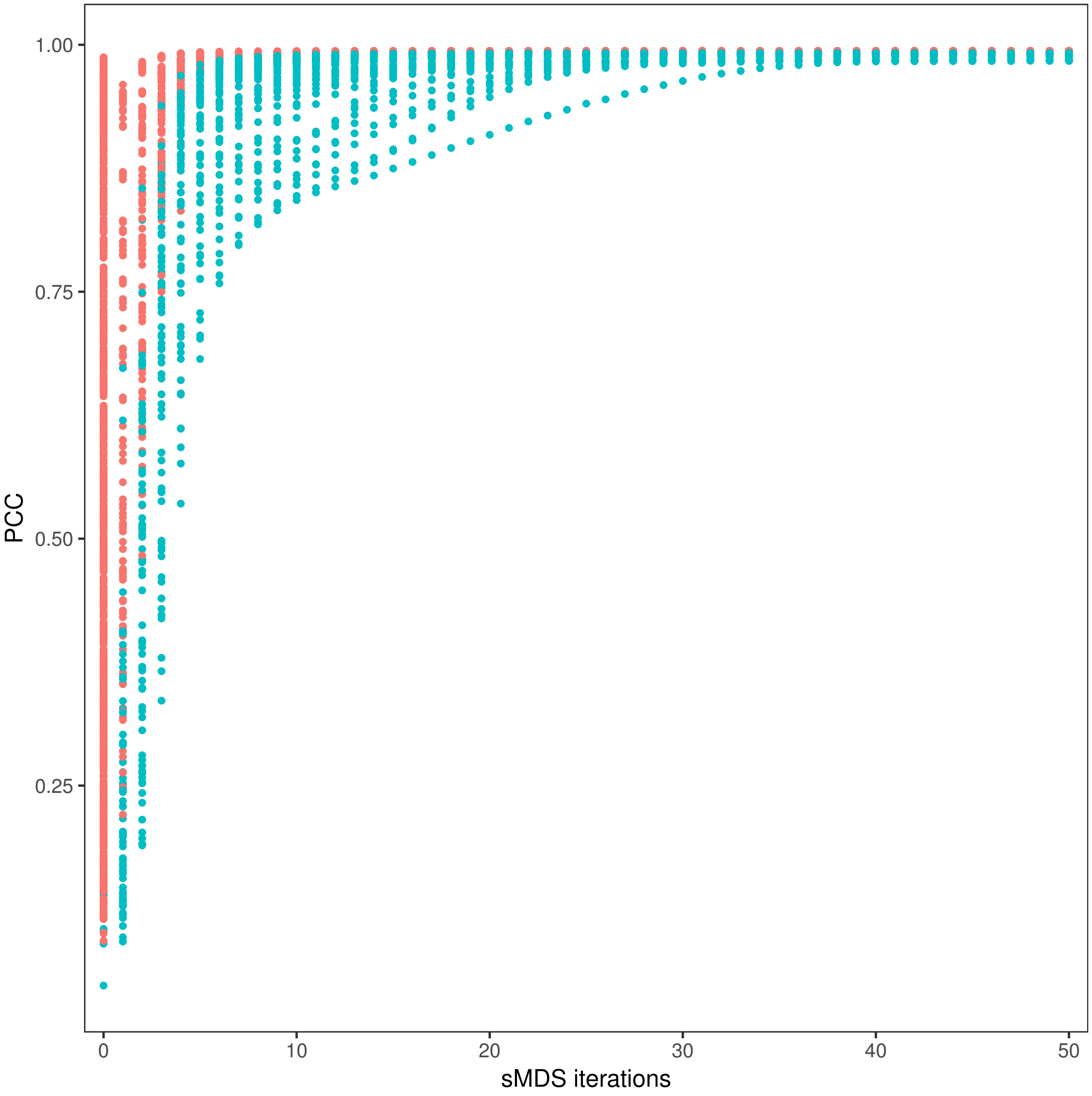

Figure S7: Comparison of reconstructed structures calculated with a different number of sMDS iterations and *in silico* structure. A combinations of different parameter settings (pivots={100,200,300,400,500,1000,2000,3000,4000,5000,10000}, overlaps={3,4,...,9,10,20,...,80,90,100}, and set size={100,200,...,900,1000,2000,3000, 4000,5000,10000}) were used, each dot on the plot corresponding to one set of parameters. Red dots corresponding to experiments with set size less than or equal to 400, and blue dots corresponding to experiments with set size larger than 400. The *in silico* structure contains 10,000 loci.

S5 Additional figures and Supplementary tables

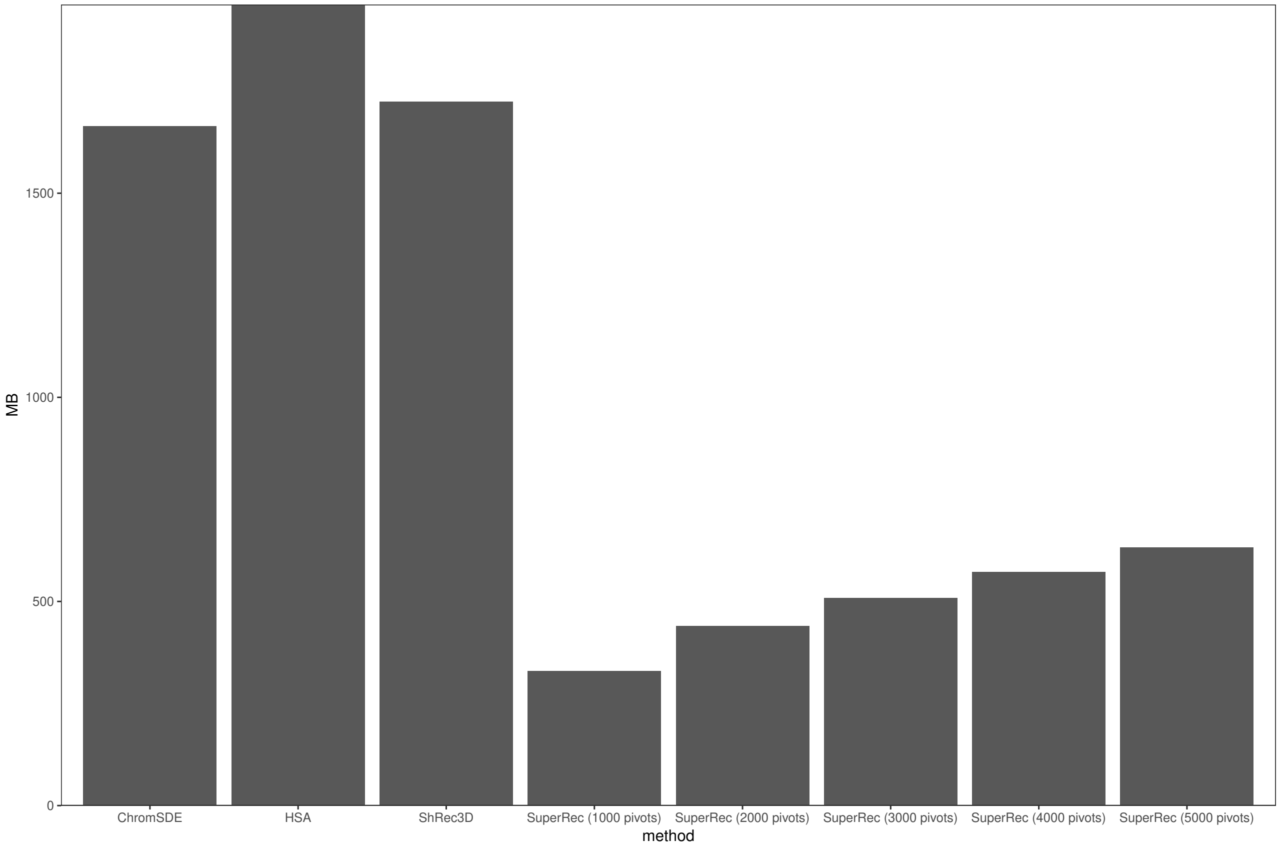

Figure S8: Comparison of memory usage when reconstructing a structure with 5,000 loci. SuperRec have a smaller memory footprint, as we increase number of pivots, SuperRec's memory usage increases as well.

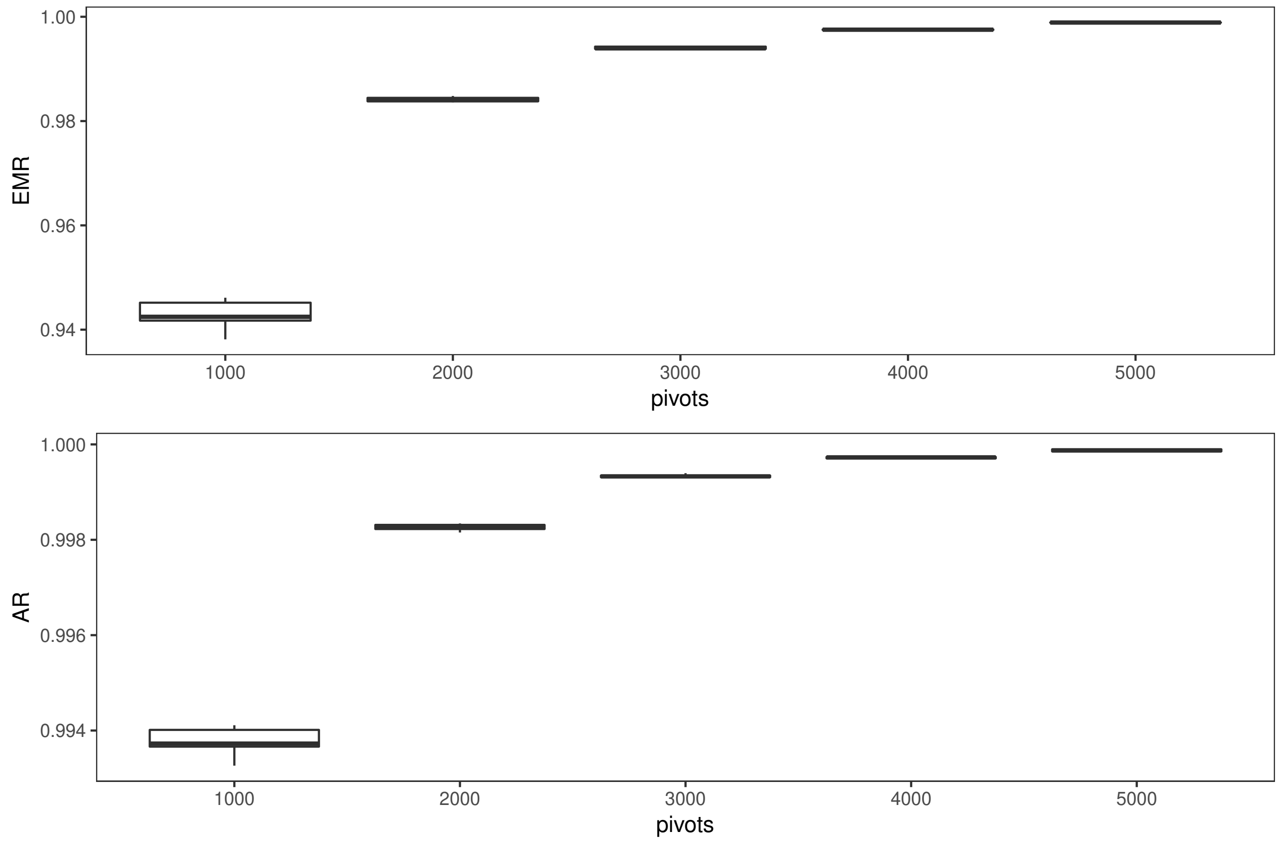

Figure S9: Box plots for EMR and AR: To access the performance of our shortest path distance approximation algorithm, different numbers of pivots were randomly selected to approximating shortest path distances of 10,000 loci. Both EMR and AR are close to 1 with very small variances, indicating the effective of our algorithm.

Table S1: *in silico* chromosomes

Loci	Structure	Loci	Structure	Loci	Structure
100	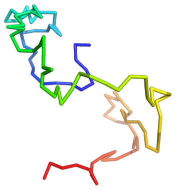	200	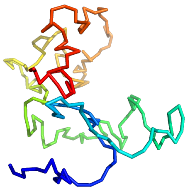	300	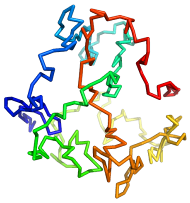
400	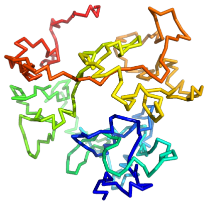	500	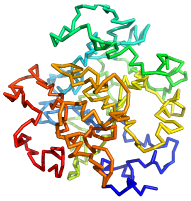	1000	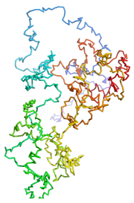
2000	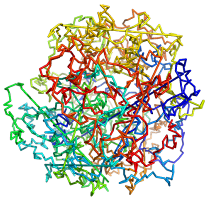	3000	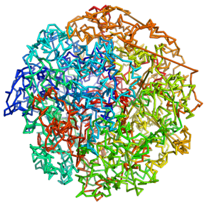	4000	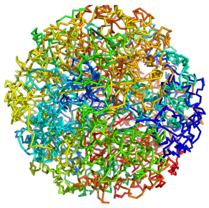
5000	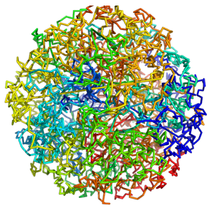	10000	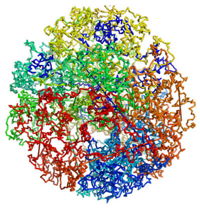	15000	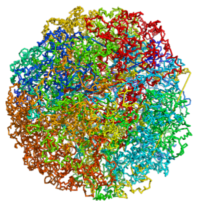
30000	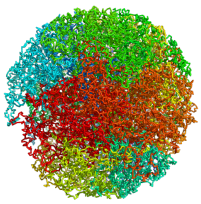				

Table S2: Comparison of reconstructed structures and regular helix structure on simulated contact matrices under various singal coverages (25%, 70%, 90%).

	25%	70%	90%
SuperRec			
	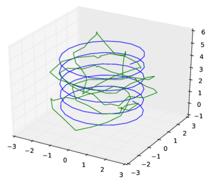	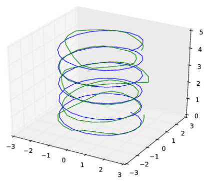	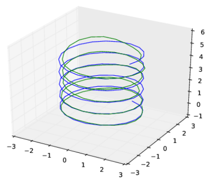
	RMSD= 0.36	RMSD= 0.13	RMSD= 0.08
ShRec3D			
	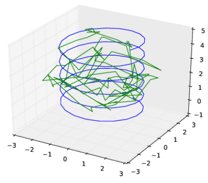	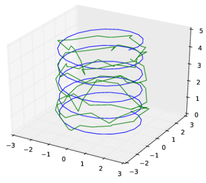	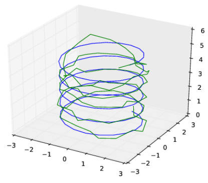
	RMSD= 0.51	RMSD= 0.21	RMSD= 0.14
HSA			
	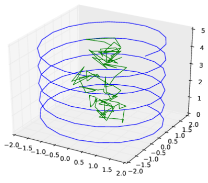	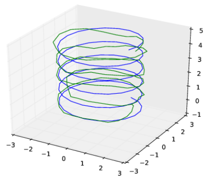	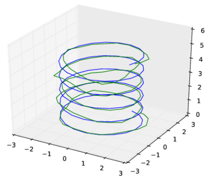
	RMSD= 0.37	RMSD= 0.15	RMSD= 0.08
ChromSDE			
	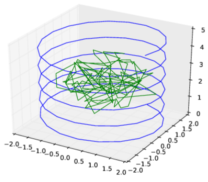	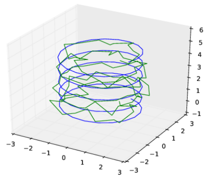	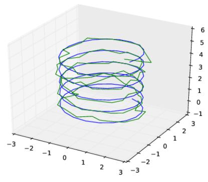
	RMSD= 1.0	RMSD= 0.20	RMSD= 0.12

References

[1] Arun, K. S., Huang, T. S., and Blostein, S. D. (1987) Least-squares fitting of two 3-D point sets. *Pattern Analysis and Machine Intelligence, IEEE Transactions on,* **PAMI-9**(5), 698–700.
